# Epigenetic Differences in Long Non-coding RNA Expression in Finnish and Russian Karelia Teenagers With Contrasting Risk of Allergy and Asthma

**DOI:** 10.3389/falgy.2022.878862

**Published:** 2022-04-27

**Authors:** Joseph Ndika, Piia Karisola, Vilma Lahti, Nanna Fyhrquist, Tiina Laatikainen, Tari Haahtela, Harri Alenius

**Affiliations:** ^1^Human Microbiome Research (HUMI), Faculty of Medicine, University of Helsinki, Helsinki, Finland; ^2^Institute of Environmental Medicine, Karolinska Institutet, Stockholm, Sweden; ^3^Department of Public Health Solutions, Finnish Institute for Health and Welfare (THL), Helsinki, Finland; ^4^Institute of Public Health and Clinical Nutrition, University of Eastern Finland, Kuopio, Finland; ^5^Skin and Allergy Hospital, Helsinki University Hospital, University of Helsinki, Helsinki, Finland

**Keywords:** long non-coding RNA, immune homeostasis, asthma, allergy, microbiota

## Abstract

**Background:**

Previously, we investigated skin microbiota and blood cell gene expression in Finnish and Russian teenagers with contrasting incidence of allergic conditions. The microbiota and transcriptomic signatures were distinctly different, with high Acinetobacter abundance and suppression of genes regulating innate immune response in healthy subjects.

**Objective:**

Here, we investigated long non-coding RNA (lncRNA) expression profiles of blood mononuclear cells (PBMC) from healthy and allergic subjects, to identify lncRNAs that act at the interphase of microbiome-mediated immune homeostasis in allergy/asthma.

**Methods:**

Genome-wide co-expression network analyses of blood cell lncRNA/mRNA expression was integrated with skin microbiota profiles of Finnish (69) and Russian (75) subjects. Selected lncRNAs were validated by stimulation of cohort-derived PBMCs and a macrophage cell model with birch pollen allergen (Betv1) or lipopolysaccharide, respectively.

**Results:**

Finnish and Russian PBMCs were differentiated by 3,818 lncRNA transcripts. In the Finnish subjects with high prevalence of allergy and asthma, a subset of 37 downregulated lncRNAs (including, *FAM155A-IT1* and *LOC400958*) were identified. They were part of a co-expression network with 20 genes known to be related to asthma and allergic rhinitis (*R* > 0.95). Incidentally, all these 20 genes were also components of pathways corresponding to *cellular response to bacterium*. The Finnish and Russian samples were also differentiated by the abundance of 176 bacterial OTU (operational taxonomic units). The subset of 37 lncRNAs, associated with allergy, was most correlated with the abundance of Acinetobacter (*R* > +0.5), Jeotgalicoccus (*R* > +0.5), Corynebacterium (*R* < −0.5) and Micrococcus (*R* < −0.5).

**Conclusion:**

In Finnish and Russian teenagers with contrasting allergy and asthma prevalence, epigenetic differences in lncRNA expression appear to be important components of the underlying microbiota-immune interactions. Unraveling the functions of the 37 differing lncRNAs may be the key to understanding microbiome-immune crosstalk, and to develop clinically relevant biomarkers.

## Introduction

Changes in the abundance and diversity of exogenous and commensal microorganisms that we encounter, are associated with immunodysregulation and incidence of allergy and asthma (1, 2). However, the mechanistic basis of the relationship between environmental stimuli, microbial diversity, faulty immune programming and the risk of asthma and allergy, is not completely understood.

The *Karelia Allergy Study* was initiated 23 years ago to study the prevalence of allergies and asthma in adjacent geographically similar regions—Russian (RUS) and Finnish (FIN) Karelia. Whilst the rural environment (cattle, domestic animals, consumption of well and spring water) has been relatively well-preserved on the RUS side, the living conditions on the FIN side of the Karelia consists of urban-type infrastructures, reduced contact with nature, and in general, a more westernized lifestyle (3). Allergies to common environmental allergens (e.g., birch pollen, timothy grass and house dust mite) and asthma were found to be more common on the FIN side (4). Skin and nasal microbiota, as well as blood leukocyte gene expression was characterized in young adults (15–20 years old) from the Finnish and Russian study participants. The resulting microbial abundance and gene expression signatures were distinct between these two populations; most notable of which was a much higher abundance of *Acinetobacter* and suppression of innate immune response genes in samples from the allergy/asthma -protected Russian subjects (5).

Long non-coding RNAs (lncRNAs) are transcripts that are over 200 base pairs long, without a significantly long open reading frame, and hence no obvious protein-coding potential. LncRNAs are the largest group of non-coding RNAs and represent up to 20% of mammalian transcriptomes. However, <3% of annotated lncRNAs have assigned functions, majority of which were identified from cancer studies (6). Relatively few lncRNAs specific to the immune system are studied. Nonetheless, from a number of well-characterized examples it is evident that lncRNAs are important modulators of the innate immune response (7). Therefore, the objective of this study was to investigate and identify functionally relevant lncRNAs involved in microbiota-mediated immune homeostasis in allergy and asthma, using existing microbiota and blood leukocyte transcriptomic data from 144 individuals in the Karelia Allergy Study cohort.

## Materials and Methods

### Study Subjects

In the *Karelia Allergy Study*, children aged 7–16 years were randomly selected from the Finnish Karelia (North Karelia, Finland; *n* = 344), and Russian Karelia (Pitkäranta, the Republic of Karelia; *n* = 427) to examine asthma and allergy prevalence (8). Teenagers and young adults (15–20 years old) from the same cohort were recruited for a follow-up study 10–12 years later, to investigate the environmental and genetic causes of contrasting asthma and allergy prevalence between the two populations (5, 9). The present study focuses only on those participants for whom skin microbiota data and peripheral blood mononuclear cells (PBMC) gene expression data are available. The study overview is shown in Supplementary Figure S1. Prevalence of pollen allergy and asthma was 1.3 and 4%, respectively, in the RUS samples (36 males and 39 females) and, 15% and 16% in the FIN samples (30 males and 39 females). Detailed clinical and demographic characteristics of these subjects have been published (5).

### Generation of Skin Microbiota and Transcriptomic Data

Sampling of skin microbial samples, DNA extraction and sequencing, has been previously described elsewhere (9). PBMCs were obtained from donors' whole blood samples and frozen until required. Thawed PBMC were left unstimulated in RPMI-1640 culture media at 37°C, and a humidified atmosphere of 5% CO_2_ for 6 and 24h, or stimulated with recombinant Betv1 in the same culture media for 6 h. Detailed description of the cell culturing, RNA isolation and microarray analysis procedures are already published (5). Only those samples with paired microbiota and transcriptomic data, where analyzed in this study: 69 FIN (Finnish Karelia) and 75 RUS (Russian Karelia) samples.

### Processing and Analysis of Microbial Data

For the microbiota data, normalized OTU (operational taxonomic unit) counts were obtained as described in Ruokolainen et al. (9). Here, normalized OTU counts were log2-transformed, and a two sample *T*-test with Benjamini-Hochberg corrected *p*-value < 0.05, was implemented to identify significantly different OTUs between the RUS and FIN samples. Principal component analysis, based on the top 2 principal components was used to visualize separation of the two populations according to significantly different OTUs. For correlation (Pearson) analysis log2-transformed relative abundance data for all significantly different OTUs were *Z*-score normalized. *Z*-score normalized OTU counts were correlated with Z-score normalized lncRNA intensity values from the same group of individuals.

### Processing and Analysis of Transcriptomics Data

Agilent's SurePrint G3 Human Gene Expression v3 Microarray Kit provides joint coverage of protein-coding genes and long non-coding RNAs in one sample. This array consists of probes against 37,756 known protein-coding transcripts as well as 30,606 non-protein coding lncRNA transcripts from both the RefSeq and LNCipedia (version 2.1) databases. Therefore, two distinct layers of the transcriptome [protein coding genes (mRNA) and long non-coding RNA (lncRNA)] can be investigated from a single microarray experiment. Changes in gene expression were analyzed with *eUTOPIA*, an *R*-based graphical user interface composed of standard bioinformatics tools for identification of differentially expressed probes, transcripts or genes (10). Probe median foreground intensities in the raw data were log2-transformed, and all probes with intensities above background signal (negative control probes) in at least half of the samples were retained in the data frame. This approach retained the majority of lncRNA transcripts for differential expression analysis since lncRNAs have an overall lower expression when compared to protein coding genes. A quantile normalization was then applied and batch effects due to labeling and array-specific variance were removed using the ComBat method (11). Transcript IDs (with NR (Refseq), XR (Refseq) or ENS (Ensembl) prefixes) were used as annotation of choice for differential expression analysis since the majority of lncRNAs do not have gene symbols. Between-group differential expression was performed by *Limma Model* analysis, using the *Benjamini-Hochberg* method for multivariate correction of false discovery rate (FDR). An FDR of at most 5% was implemented as cut-off to consider a transcript as significantly differentially expressed between RUS and FIN samples. Coding (mRNA) and non-coding (lncRNA) genes were separated into two data layers using their RefSeq and/or ENSEMBL identifiers.

### Identification of Functionally Relevant LncRNA

LncRNAs can regulate gene expression by directly binding to DNA/RNA, or indirectly, by being miRNA precursors or miRNA “sponges” (12). To identify lncRNAs that potentially drive microbiota-mediated mechanisms associated with contrasting allergy and asthma prevalence, three data layers were generated from all study subjects: mRNA (protein-coding genes only), lncRNA (long non-coding RNA) and skin microbiota abundance profiles. We then used pathway enrichment analysis and correlation-based filtering to:

i) generate a *lncRNA:mRNA* co-expression network consisting of lncRNA transcripts that corelate (Pearson's *R* > 0.95) only with differentially expressed (RUS vs. FIN) mRNA genes that are enriched in biological pathways corresponding to cellular response to microbes and their components.
ii) to identify a *lncRNA:OTU* co-abundance network consisting of microbial OTUs with the strongest correlation to those lncRNAs that are co-expressed with microbe response genes.

Biological pathway enrichment analysis was performed using Gene Ontology's *Panther* tool, which determines overrepresented biological process categories from a list of input genes (13). To enhance the phenotypic relevance of the identified pathways only mRNA genes with a fold-change < 1.5 (log2 difference >0.58) in RUS/FIN samples were retained for pathway enrichment analysis. No fold change cut-offs were applied to differentially expressed lncRNAs or OTUs, because they may significantly affect the abundance of protein-coding genes via a plethora of different mechanisms that do not necessarily rely on big changes in their expression (lncRNA) or abundance (OTU). The *Perseus* bioinformatics data analysis suite (14) was used for integration (transformation, Z-score normalization, correlation analysis and heatmaps) of the three (lncRNA, mRNA, OTU) data layers. Pearson correlation across data layers was performed on Z-score normalized data, after log2 transformation. The clustering parameters for heatmaps were as follows: Distance: Euclidean, Linkage: Average, and Cluster Pre-processing: K-means.

### Validation of LncRNA Expression and Activity

Selected top differentially expressed lncRNAs identified as key components of microbiota-associated mechanisms in asthma and allergy, were validated by gene expression analysis of differentiated THP1 cell lines stimulated with lipopolysaccharide (LPS), and cohort-derived PBMC cultures stimulated with recombinant Betv1 protein. THP1 cell lines (ATCC, Rockville, MD, USA) are human leukemia monocytic cells. They were grown in suspension in RPMI supplemented with 10% FBS, 1% GlutaMAX, 1% HEPES, 0.05 mM 2-ME, and 1% PEST at 37°C and 5% CO_2_ until densities of 0.2 to 1.5 x 10^6^ cells/mL. The cells were then transferred to fresh cell culture medium supplemented with 50 nM differentiated phorbol-12-myristate-13-acetate (PMA), for 48 h to trigger macrophage differentiation. This differentiation medium was refreshed once after the first 24 h. Differentiated THP1 cells were incubated for 24 h with 10 ng/mL purified LPS (*Escherichia coli* 0111: B4, Sigma-Aldrich). Untreated cells in culture media were taken along as controls. Cell passages used were between 7 and 10. RNA was isolated using the Total RNA Purification Plus Kit (Norgen Biotek, Canada). Five hundred nanogram of total RNA was used for cDNA synthesis (High-Capacity cDNA Reverse Transcription Kit; ThermoFisher Scientific). Primers and probes for *LOC731464* (MIR3945HG), *LINC01197, IL1B*, and *IL6* were ordered as pre- designed Taqman Gene Expression Assays (ThermoFisher Scientific). Real-time amplification was performed with TaqMan's Fast Advanced Master Mix in 96-well optical reaction plates on a standard 7500 Fast RT-PCR system (Applied Biosystems). Expression of 18S rRNA was used as an endogenous control to account for technical variations during sample prep.

Donor-derived PBMC were obtained and treated with recombinant Betv1 (10 μg/ml, Indoor Biotechnologies, Product Code RP-BV1-1) for 6 h as described in *generation of skin microbiota and transcriptomic data* above. Control samples were incubated in untreated cell culture media for 6 h. Gene expression analysis of control and Betv1-stimulated PBMC was done by microarray, as described above. Expression of selected mRNA and lncRNA in Finnish subjects with birch pollen allergy was obtained from the normalized and batch-corrected data matrix.

## Results

### LncRNA, mRNA and Skin Microbiota Profiles, Are Distinct Between the Finnish and Russian Subjects

A total of 3818 lncRNA transcripts, 6332 mRNA genes and 176 skin bacterial OTUs were identified as significantly different (FDR < 0.05) between RUS and FIN subjects. All three data layers, without pre-defined fold-change cut-offs, clearly separated FIN from RUS samples (Supplementary Figure S2). Visualization of the top two principal components revealed that majority (41%) of the variance between the two populations was explained by differences in mRNA expression, followed by 35% variance due to lncRNA expression and 22% from differences in their microbiota abundance profiles.

### Fifty-Four LncRNAs Are Co-expressed With 73 mRNA Genes Involved in Microbial Immunity

Since the biological functions of most lncRNAs are unknown, we hypothesized that lncRNAs whose expression strongly corelates with specific functional hubs of mRNA genes, are most likely regulators of the biological function performed by these genes. Due to the fact that a clear distinction has been observed between the microbiota of allergy prone and allergy-protected individuals in this cohort, our specific objective was to identify lncRNA that potentially regulate genes that are known to be involved in cellular response to microbes.

**Figure 1:**
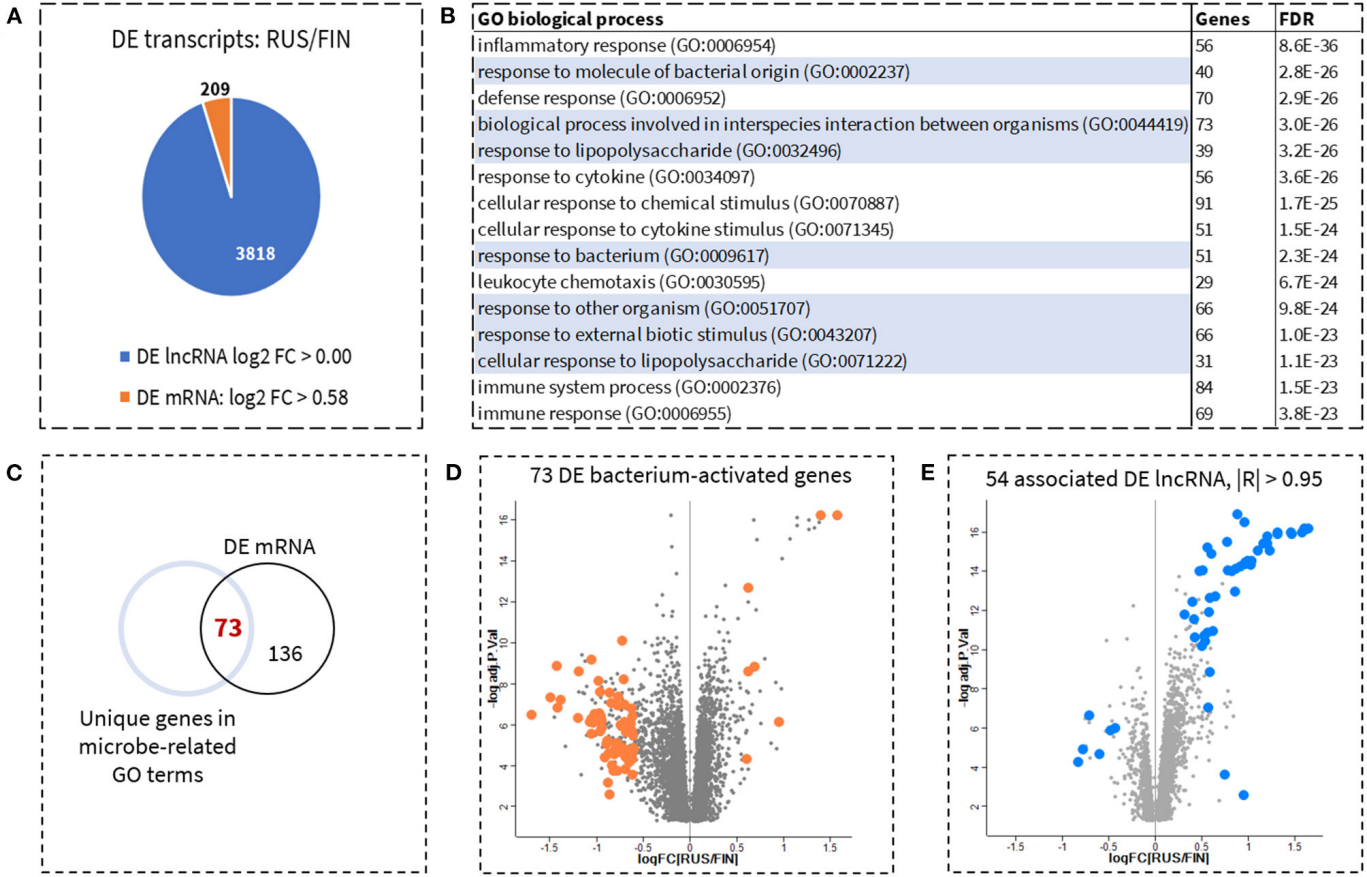
Identification of genes involved in cellular response to microbes and prediction of upstream regulator lncRNAs. Six thousand three hundred thirty-two mRNA genes and 3,818 lncRNA transcripts, were significantly different between the Russian (RUS) and Finnish (FIN) populations. To prioritize identification of biologically relevant mRNA, only genes with a fold-change >1.5 (log2 difference > 0.58) were retained for pathway enrichment analysis. No fold-change filtering was implemented for the lncRNAs. This resulted in a total of 209 DE mRNA and 3,818 DE lncRNA **(A)**. Gene Ontology (GO) based functional enrichment analysis with the 209 mRNA as input genes, revealed several biological processes corresponding to inflammatory response, most notable of which were cellular response to microbial stimuli. Top enriched biological processes (sorted by adjusted *p*-value) including highlighted biological processes related to cellular response to bacteria are shown in **(B)**. In total 73 unique genes, were identified across all microbe-associated pathways **(C)**. A volcano plot of these 73 microbe-associated mRNA genes shows that these are some of the top DE genes in RUS vs. FIN samples [**(D)**, orange dots]. Fifty-four lncRNAs were identified as highly significantly correlated (Pearson's |*R*| > 0.95) to at least 1 of the 73 microbe-associated genes. This hub of 54 lncRNAs are also some of the top DE lncRNA transcripts between RUS and FIN blood samples [**(E)** blue dots].

In total, 6,332 mRNA genes were differentially expressed (DE) between RUS and FIN samples. Two hundred and nine mRNA genes had a fold change in expression that exceeded 1.5-fold. To focus on the most prominent gene-related functions associated with allergy and asthma prevalence, we selected only those genes with more than 1.5-fold change in expression between RUS and FIN samples (Figure 1A) for pathway enrichment analysis. No fold-change cut-off was applied to DE lncRNA transcripts. The 15 most significantly enriched (FDR < 4.0 E-23) biological processes, consisting of 29–91 mRNA genes, are shown in Figure 1B. Seven of the top 15 biological processes correspond to cell-mediated immune response to microbes and their components (highlighted in Figure 1B). All enriched mRNA genes from the seven different gene ontology (GO) categories were pooled together. Venn analysis revealed a total of 73 unique mRNA genes with GO annotation; *biological process involved in the interspecies interaction between organisms* (GO: 0044419, FDR = 3.0 E-26) (Figure 1C). A scatterplot of these 73 genes, shows that they are amongst the top DE mRNA genes (by fold-change) between RUS and FIN blood cell samples, 90% of which (66 genes) were downregulated in the RUS samples (Figure 1D). Pearson's correlation analysis shows that 54 of the 3,818 DE lncRNA transcripts were highly significantly correlated (|R| > 0.95) to at least 1 of the 73 mRNA genes: which we herein refer to as the *54/lncRNA:73/mRNA* co-expression network. Similar to the mRNA genes, a scatter plot of the 54 corelated lncRNA transcripts shows that they are also amongst the top DE transcripts in RUS vs. FIN blood cell samples. However, the direction of change in expression was mostly opposite to that of the mRNA genes, with 48 lncRNA transcripts upregulated in RUS compared to FIN blood cell samples (Figure 1E). A list of all 54 corelated lncRNAs, with their corrected *p*-values and fold-change in expression between RUS and FIN, is provided in Supplementary Table S1.

### The Co-expressed LncRNA:mRNA Network Constitutes 20 Genes That Are Involved in the Pathophysiology of Allergic Asthma and/or Allergic Rhinitis

**Figure 2:**
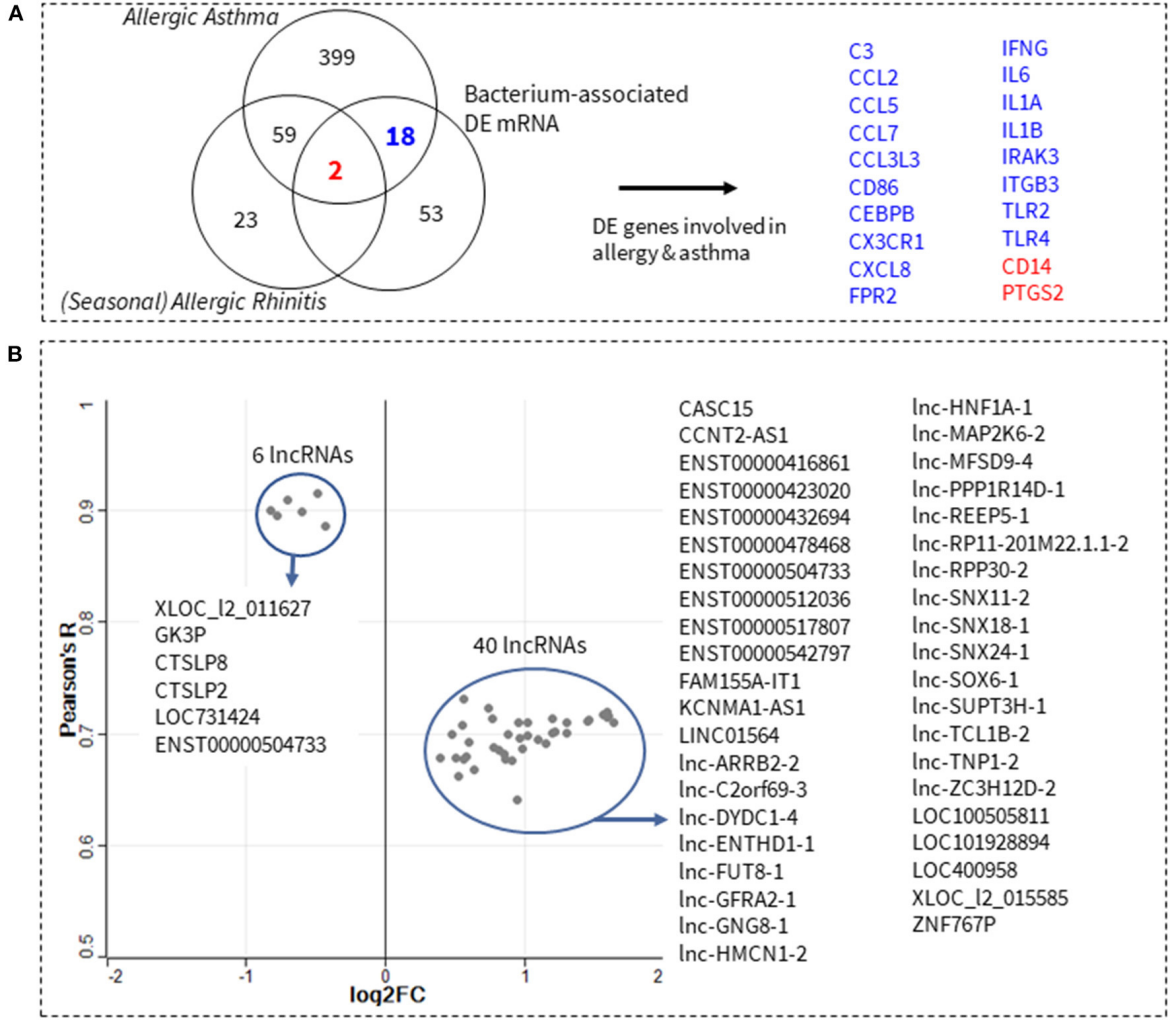
Prediction of lncRNAs involved in allergy and asthma pathophysiology. Known allergic rhinitis and allergic asthma genes were downloaded from Ingenuity's Knowledgebase (IPA, QIAGEN). Venn comparisons show 20 of these genes overlap with the 73 microbe-associated genes from this study **(A)**. In total, 47 differentially expressed (DE) lncRNA transcripts (log2 difference > 0.4, adjusted *p*-value < 0.002) were found to be significantly correlated (Pearson's |*R*| > 0.95, *p*-value < 0.05) to at least one of the 20 genes in **(A)**. A scatter plot of the median correlation of these 47 lncRNA transcripts to all 20 allergic asthma/rhinitis genes, against their fold-change in expression (log2) between Russian and Finnish samples is shown in **(B)**. Gray dots correspond to each of the 47 lncRNAs, while blue circles show the 6 and 40 lncRNAs that are downregulated and upregulated, respectively.

To further refine the functional relevance of the identified *54/lncRNA:73/mRNA* co-expression network, we next sought to answer whether any of its component mRNA genes are involved in allergic rhinitis or allergic asthma. Five hundred and one allergic asthma and allergic rhinitis genes were identified and downloaded from Ingenuity's Knowledgebase (IPA, QIAGEN). Venn comparisons showed an overlap of 20 genes with the 73 genes in the *54/lncRNA:73/mRNA* co-expression network (Figure 2A). Next, we filtered the *54/lncRNA:73/mRNA* co-expression network, such that only the 20 overlapping genes and their corresponding correlated (|R| > 0.95) lncRNA transcript pairs were retained in the lncRNA:mRNA co-expression network. This resulted in a smaller network consisting of 46 lncRNA transcripts and 20 allergic asthma and allergic rhinitis genes—which we herein called the *46/lncRNA:20/mRNA* co-expression network. The importance of each lncRNA in the co-expression network, was inferred from its average correlation to all genes in the network. A scatter plot of the median absolute correlation of the 46 lncRNAs to all 20 asthma and allergic rhinitis genes, as well as the fold-change in lncRNA expression between RUS and FIN samples, is shown in Figure 2B. All 46 lncRNAs were found to have a robust association (Median R > 0.63) with the network of 20 asthma and allergic rhinitis genes. Six lncRNAs in particular (*XLOC_l2_011627, GK3P, CTSLP8, CTSLP2, LOC731424*, and *ENST00000504733*), had a very strong median correlation of 0.88 to 0.92. These 6 lncRNAs were downregulated in RUS blood cell samples, while the remaining 40 correlated lncRNAs (median *R* of 0.63–0.75) were upregulated.

**Figure 3:**
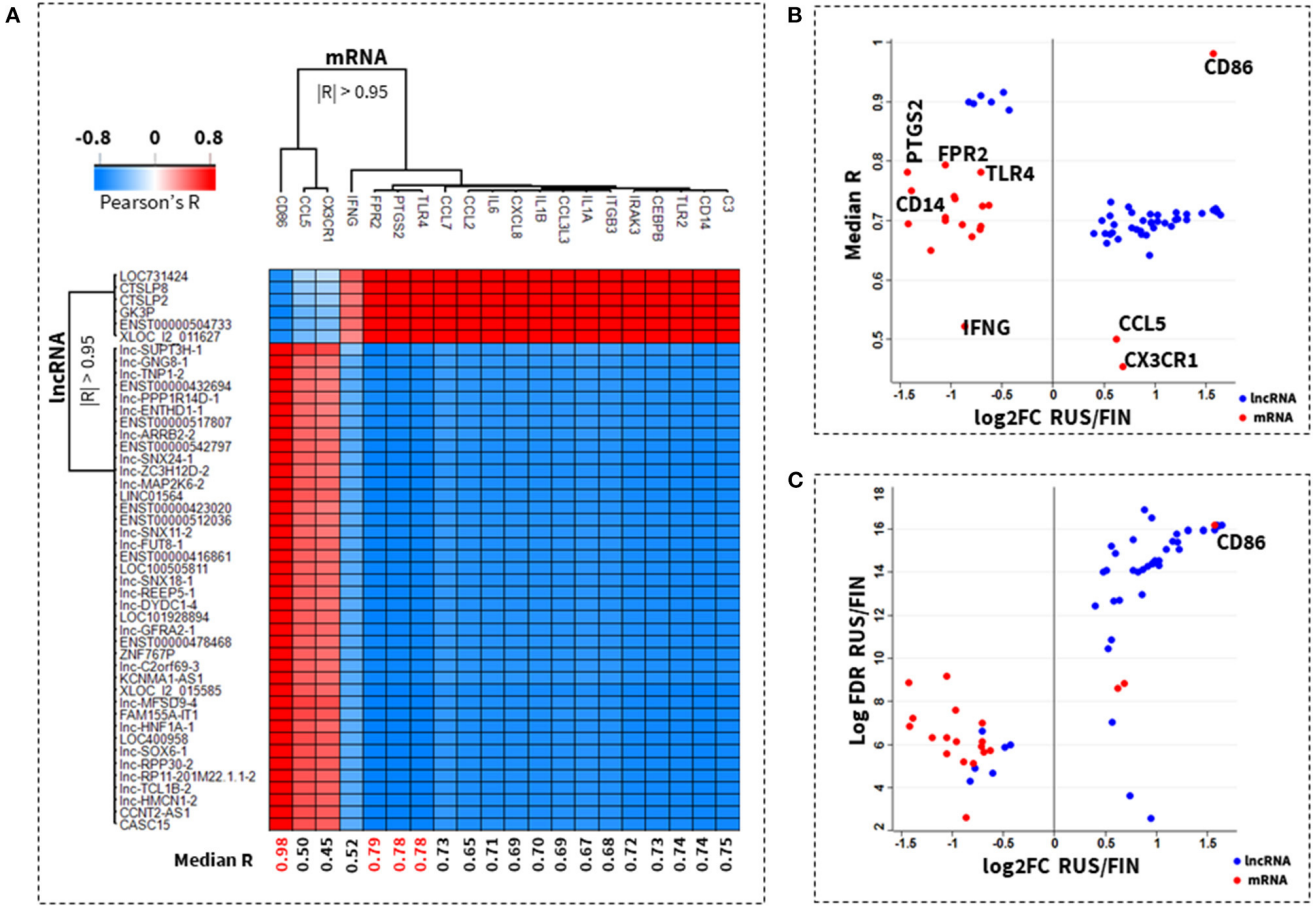
Co-expressed lncRNA-mRNA network with top ranked key genes. A correlation score heatmap of 47 lncRNAs with 20 known allergic rhinitis and allergic asthma mRNA genes, is shown in **(A)**. The importance of an mRNA gene was inferred from its average correlation (median R) to all participating lncRNAs in the network. A plot of the median correlation against the fold-change (log2) in expression between Russian and Finnish samples is shown in **(B)**. The top ranked genes are *CD86, FPR2, PTGS2, TLR4*, and *CD14* and the lowest ranked genes are *CX3CR1, CCL5, IFNG*. A plot of the fold-change in expression against their log-transformed *p*-values, shows that the lncRNA transcripts in the 47/lncRNA:20/mRNA co-expression network have the most significant difference in expression between Russian and Finnish samples, when compared to the mRNA genes **(C)**.

The importance of each mRNA gene in the *46/lnRNA:20/mRNA* network was also inferred from its average correlation to all lncRNA transcripts in the same network. A correlation score heatmap of each gene-lncRNA pair in the *46/lnRNA:20/mRNA* co-expression network is depicted in Figure 3A. *CD86* (median *R* = 0.98), *FPR2* (median *R* = 0.79), *PTGS2* (median *R* = 0.78) *TLR4* (median *R* = 0.78), and *CD14* (median *R* = 0.74) were identified as the most important genes in the *46/lnRNA:20/mRNA* co-expression network. Meanwhile, *IFNG* (median *R* = 0.52), *CCL5* (median *R* = 0.50), and *CX3CR1* (median *R* = 0.45) had the weakest average correlation (Figures 3A,B).

Also, the lncRNA were almost entirely upregulated in RUS samples, and had a more significant change in expression in RUS vs. FIN samples when compared to the change in mRNA expression between these two groups. Majority (17 out of 20) of the mRNA on the other hand were predominantly downregulated in RUS blood cell samples. Only the CD86 gene had a change in expression that was comparable in significance and directionality (upregulated) to the topmost differentially expressed lncRNAs in the *46/lnRNA:20/mRNA* network. In Figure 3C, a scatterplot of the ranked (log-transformed) *p*-values vs. the fold-change in expression between RUS and FIN samples is shown for the genes in the *46/lnRNA:20/mRNA* network.

### Asthma-Protective Acinetobacter Is the Top Positively Correlated OTU, With the 46/LncRNA:20/mRNA Co-expression Network

**Figure 4:**
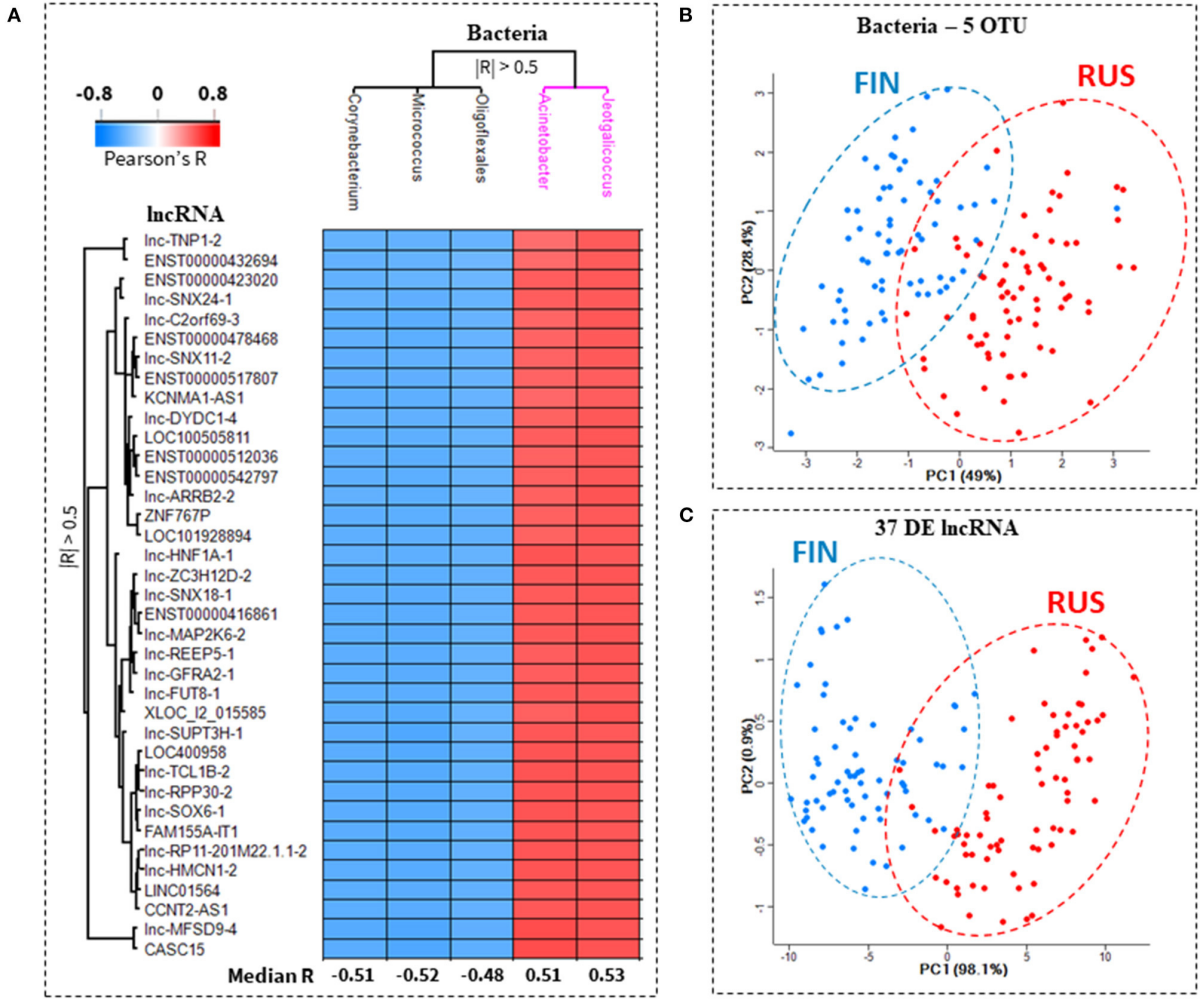
Top ranked bacterial OTUs associated with expression of key lncRNA transcripts. A heatmap of the correlation between predicted microbe-associated lncRNAs and skin microbiota in Russian and Finnish subjects, is shown in **(A)**. Thirty-seven lncRNA transcripts were significantly correlated (Pearson's |*R*| > 0.05, *p*-value < 0.05) to at least 1 out of and 5 bacterial OTUs (operational taxonomic unit). The lncRNAs were correlated to two separate clusters of bacteria. In cluster 1 (black), all 37 lncRNA transcripts were anti-correlated with the abundance of Corynebacterium, Micrococcus and Oligoflexales. In cluster 2 (purple), all 37 lncRNA transcripts were positively correlated with the abundance of Acinetobacter and Jeotgalicoccus **(A)**. Principal component analysis based exclusively on either the relative abundance of the 5 bacterial OTU **(B)**, or the relative expression of these 37 lncRNA transcripts **(C)**, clearly separates the Finnish (FIN, blue dots) from the Russian (RUS, red dots) subjects.

We next sought to determine which of the differentially abundant (RUS/FIN) microbial OTUs, have the strongest correlation to lncRNA transcripts within *the 46/lncRNA:20/mRNA* co-expression network. At a threshold of |R| > 0.5, *p*-value < 0.05, 5 bacterial OTUs and 37 lncRNA transcripts were retained in the Pearson's correlation matrix. Microbial abundance of *Acinetobacter* and *Jeotgalicoccus* genera, were both positively correlated to all 37 lncRNA transcripts, while the abundance of *Corynebacterium* and *Micrococcus* genera were negatively correlated to all 37 lncRNA transcripts. One unclassified OTU from the Order *Oligoflexales* was also negatively correlated to all 37 lncRNA transcripts. A heatmap of the correlation scores within this *37/lncRNA:5/OTU co-abundance* network, and, the median correlation of each OTU to all 37 lncRNAs, is shown in Figure 4A. Principal component analysis, based either on the relative abundance of the 5 lncRNA-associated OTUs (Figure 4B) or relative expression of the 37 OTU-associated lncRNAs (Figure 4C) separates the RUS and FIN subjects in this cohort.

### Validation of Differentially Expressed *FASM155A-IT1, LOC400958*, and LOC731424

**Figure 5:**
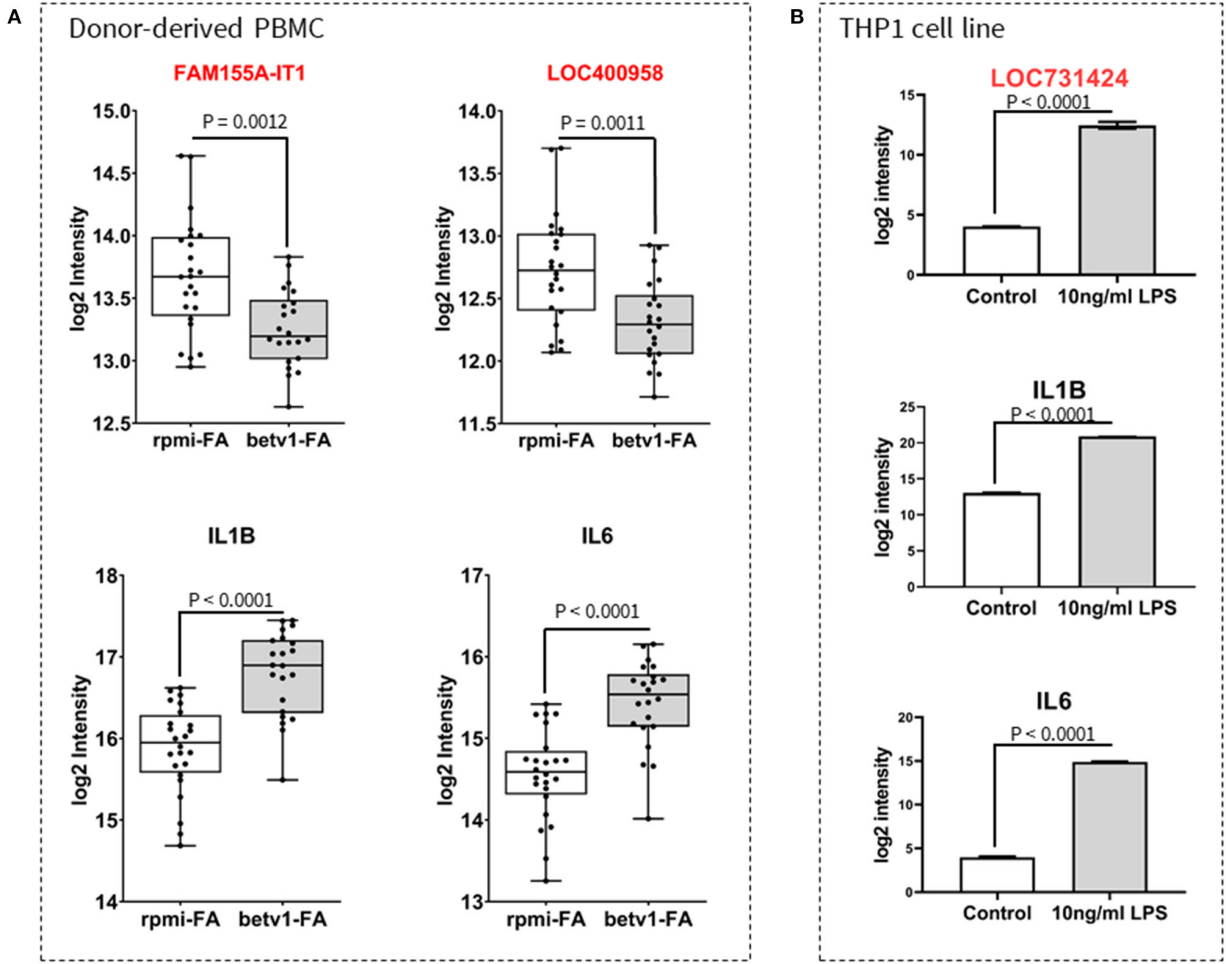
Validation of lncRNA expression and response to innate/adaptive immune stimuli. Expression of selected lncRNA and cytokine genes in peripheral blood mononuclear cells (PBMC) cultures **(A)** and a macrophage-like THP1 cell line **(B)** are shown. PBMCs were isolated from whole blood of individuals diagnosed with birch allergy, and stimulated for 6 h with recombinant Betv1 pollen allergen. Unstimulated cells were taken along as controls. Similar to pro-inflammatory cytokines (*IL1B* and *IL6*), there was a significant change in the expression of *FAM155A-IT1* and *LOC400958* **(A)**. However, whilst *FAM155A-IT1* and *LOC400958* were both downregulated in response to Betv1 stimulation [**(A)**, upper panel], *IL1B* and *IL6* were upregulated in allergen-stimulated PBMC from allergic FIN subjects [**(A)**, lower panel]. In THP1 cells, LPS stimulation caused an increase in expression of *LOC731424, IL1B* and *IL6* **(B)**.

To further explore whether the identified allergy/asthma- and microbe-associated lncRNAs are involved in innate/adaptive immune responses, we investigated the expression of three lncRNA transcripts identified in both the *46/lncRNA:20/mRNA* and *37/lncRNA:5/OTU* networks. A second set of PBMCs isolated from whole blood of FIN subjects with birch pollen, were cultured for 6 hours in either RPMI (unstimulated controls) or RPMI + Betv1 (allergen-stimulated). *FAM155A-IT1* and *LOC400958* expression were significantly downregulated after Betv1 stimulation (Figure 5A, upper panel), while the gene expression of pro-inflammatory IL1B and IL6 was significantly increased in Betv1-stimulated cells when compared to unstimulated controls (Figure 5A, lower panel). To study the response of the innate immunity ligand on lncRNA gene expression, differentiated THP1 cells were cultured and stimulated with toll-like receptor (TLR) 4 agonist LPS for 24 h. Expression of *LOC731424, IL1B*, and *IL6*, were all significantly upregulated when compared to unstimulated control cells (Figure 5B).

## Discussion

Allergen sensitization, allergic rhinitis and asthma are intertwined, especially through childhood (15). The natural environment influences human commensal microbiota composition, and as a result, their ability to guide antigen-recognizing receptors to enhance, terminate or prevent inappropriate inflammatory responses (16). In our previous studies we have shown that high *Acinetobacter* abundance is associated with reduced allergy outcome (17), and that intradermal application of *Acinetobacter*, inhibited allergen sensitization and development of lung inflammation in mice (18). Thus far, transcriptomics has been extremely valuable in advancing our understanding of asthma endotypes and mechanisms of disease. However, the role of lncRNAs in allergy and asthma is as yet understudied.

LncRNAs can effectuate stable or repressive chromatin states, to increase or decrease transcriptional activation, physically interact with DNA, RNA or proteins to alter the stability and translation status of mRNAs, and they can be miRNA precursors or contain binding sites to sequester miRNAs from their mRNA targets (12). LncRNAs have been identified in almost all immune cells (19). Due to their low evolutionary conservation and heterogenous modes of action (12, 20), determining the specific functions of lncRNAs is challenging. Since lncRNAs ultimately affect gene expression in diverse physiological and pathological contexts, we hypothesized that construction of lncRNA:mRNA co-expression networks is a more straightforward approach to predict upstream lncRNAs that regulate specific subsets of genes. Co-expressed genes often share similar functions (21), and as such correlation-based co-expression networks have been used to determine and validate the functions of several lncRNAs (22). Also, to integrate the effect of environmental microbial exposures (microbiota) on lncRNA expression and function, we performed the correlational analysis to mRNA genes from *cellular response to microbe* pathways. Even though we did not implement any fold-change cut-offs, the lncRNA genes within the *54/lncRNA:73/mRNA* co-expression network had a fold-change in expression (RUS/FIN) of 1.2- to 3-fold (Supplementary Table S1). The fact that 20 known allergic asthma and allergic rhinitis genes were identified in the subset of 73 microbe-responsive genes, further highlights the interconnected relationship between the microbiota and asthma/allergy. Furthermore, 46 lncRNAs were strongly co-expressed (*R* > 0.95) with these 20 known asthma/rhinitis genes.

We postulate that these lncRNA genes represent a hub of non-protein-coding transcripts involved in driving an immune tolerance network that defines microbiota-mediated predisposition to allergy/asthma. This is additionally supported by the finding that 80% (37 out of 46) of these lncRNAs were most associated (|*R*| > 0.5) with the abundance of 4 of the top bacterial OTUs that separate Russian from Finnish samples with contrasting prevalence of allergy/asthma (9). The hub of 37 lncRNAs were all positively correlated with *Acinetobacter* and *Jeotgalicoccus* (more abundant on skin and nasal epithelium of Russian subjects with low prevalence of allergy/asthma), and they were also all negatively correlated to *Corynebacterium* and *Micrococcus* (more abundant on skin and nasal epithelium of Finnish subjects with low prevalence of allergy/asthma). Several other epidemiological and experimental studies have also replicated the protective roles of the *Acinetobacter* and/or *Jeotgalicoccus* genera. For example, in 489 school-age children from rural and sub-urban Germany, abundance of *Jeotgalicoccus* sp. and *Acinetobacter* sp. were inversely associated with asthma, atopic sensitization and hay fever (23). Also, intradermal exposure of an asthmatic mouse model to *Acinetobacter lwoffii* conferred protection against allergic sensitization and lung inflammation (18). And lastly, reversal of eosinophilic and neutrophilic airway inflammation was demonstrated in allergic asthma mouse models after nasal treatment with *Staphylococcus sciuri*—a gram-positive bacteria belonging to the same Family (Staphylococcaceae) as *Jeotgalicoccus* (24).

A recurring theme from existing mechanistic studies is that the protective role of *Acinetobacter* derives from suppression of pro-inflammatory mediators. It is plausible that this is regulated by activation of a specific subset of lncRNAs, such as the hub of 37 lncRNAs identified herein. The most negatively correlated genes within the lncRNA:mRNA co-expression network played a role in prostaglanding biosynthesis (*PTGS2*), monocyte chemotaxis (*FPR2*) and LPS recognition (*TLR4*), all of which are known to enhance inflammatory responses. These genes were downregulated in RUS samples (compared to FIN) with high *Acinetobacter* abundance and low allergy/asthma prevalence. A key upregulated gene from the lncRNA:mRNA co-expression network was CD86—the major co-stimulatory molecule on antigen-presenting cells. The pathways and interactions that explain how lncRNAs regulate adaptive immune responses are not clear, but switching of dendritic cells toward a tolerant phenotype and promotion of regulatory T cell differentiation are suggested (25, 26). At the very least, our experimental findings that (1) *LOC731424* expression increases in response to microbial component (LPS) in antigen presenting cells and (2) expression of *FAM155A-IT1* and *LOC400958* decrease in allergen (Betv1) stimulated immune cells from allergic patients, in a manner that was comparable or antagonistic to the change in expression of pro-inflammatory IL1B and IL6 cytokines, confirms that some of these lncRNAs are involved in immune response signaling.

A general limitation of this study relates to the fact that the majority of our lncRNA findings are primarily based on correlative evidence. This was mitigated by using very robust correlation cut-offs (*R* > 0.95) to determine lncRNA:mRNA co-expression networks, followed by verification of selected hub lncRNAs in a macrophage-like cell line model and stimulated donor-derived primary cells. The relevance of our findings to the studied phenotype (contrasting prevalence of allergy/asthma), is highlighted by the fact that principal component analyses based only on the identified core subset of 37 lncRNAs or their top associated microbiota (5 bacterial OTUs), clearly distinguishes the Finnish and Russian subjects in this cohort. Subsequent mechanistic studies, targeting some of the transcripts from the hub of 37 lncRNAs will be needed to further elucidate the mechanistic basis of microbe-lncRNA crosstalk in immune homeostasis.

## Conclusion

Previously, an asthma/allergy-protective role of the microbiome was demonstrated in Russian subjects with low asthma/allergy prevalence, relative to their Finnish counterparts. In this study, we identified 3,818 lncRNA transcripts that differentiate the two sample groups in this cohort. By integrating mRNA and lncRNA expression profiles, we identified a network of co-expressed lncRNA transcripts that are associated with microbe-responsive genes. Furthermore, by retaining only lncRNAs with a strong association to known asthma and allergic rhinitis genes, a refined hub of 37 lncRNAs involved in microbe-immune interactions in asthma and allergy was established. We propose that, upregulation of the identified subset of 37 lncRNA genes, provides the mechanistic link to the asthma/allergy-protective role of gram-negative *Acinetobacter* and gram positive *Jeotgalicoccus* bacteria in skin and nasal microbiota. Our findings open up new and exciting avenues to further elucidate the relevance of lncRNA expression in immune homeostasis. For example, whether the identified lncRNA signature is stable within and outside the allergy season, and also if this signature is sustained in adults with chronic asthma. Expression profiling of these lncRNAs could also be used as epigenetic signatures to investigate beneficial or deleterious microbiota-immune interactions in asthma and allergy.

## Data Availability Statement

The original contributions presented in the study are included in the article/Supplementary Materials, further inquiries can be directed to the corresponding author/s.

## Ethics Statement

Ethical review and approval was not required for the study on human participants in accordance with the local legislation and institutional requirements. Written informed consent to participate in this study was provided by the participants' legal guardian/next of kin.

## Author Contributions

JN: study concept, data analysis, data interpretation, figures, and writing—original draft preparation. PK: data generation, data interpretation, figures, and writing—review and editing. NF: data generation and writing—review and editing. TL: cohort design, data collection, and writing—review and editing. TH: cohort design, data interpretation, and writing—review and editing. HA: study concept, cohort design, data interpretation, figures, and writing—review and editing. All authors have read and agreed to the published version of the manuscript.

## Funding

This work was supported the following grants from KONE foundation (grant number: 201801011), the Swedish Research Council (grants 2017-01373 and 2020-02090), Academy of Finland (decision number 338325), and the Finnish Cultural Foundation.

## Conflict of Interest

The authors declare that the research was conducted in the absence of any commercial or financial relationships that could be construed as a potential conflict of interest.

## Publisher's Note

All claims expressed in this article are solely those of the authors and do not necessarily represent those of their affiliated organizations, or those of the publisher, the editors and the reviewers. Any product that may be evaluated in this article, or claim that may be made by its manufacturer, is not guaranteed or endorsed by the publisher.

## Supplementary Material

Supplementary Figure S1 Study design. Skin swab and whole blood samples derive from 69 Finnish and 75 Russian subjects, with marked differences in the prevalence of asthma and allergies. All 144 study subjects had paired skin microbiota data from skin swabs and transcriptomic data from peripheral blood mononuclear cells (PBMC). Correlation to protein-coding gene (mRNA) expression and microbiota abundance was used to predict long non-coding RNAs (lncRNA) involved in microbiota-immune interactions in asthma and allergy. Selected lncRNAs were validated with lipopolysaccharide and Betv1 allergen stimulations in a macrophage-like cell model (THP1) and donor-derived PBMC.

Supplementary Figure S2 Gene and microbiota-based distinction of Finnish and Russian samples. Principal component analysis of significantly different (adjusted *p*-value < 0.05) long non-coding RNAs [**(A)**: lncRNA], protein-coding genes [**(B)**: mRNA] and skin microbial taxa [**(C)**: OTU]. All three data layers separate Finnish (FIN) from Russian (RUS) samples. In principal components 1 and 2, the most variation (41%) between the two populations was explained by differences in mRNA expression, followed by lncRNA expression (35%) and then microbiota abundance profiles (22%).
