# Supplementary figures and images for: Epigenetic Differences in Long Non-coding RNA Expression in Finnish and Russian Karelia Teenagers With Contrasting Risk of Allergy and Asthma
Source: Front Allergy. 2022 Apr 27;3:878862. doi: 10.3389/falgy.2022.878862 (PMC9234912; doi:10.3389/falgy.2022.878862)

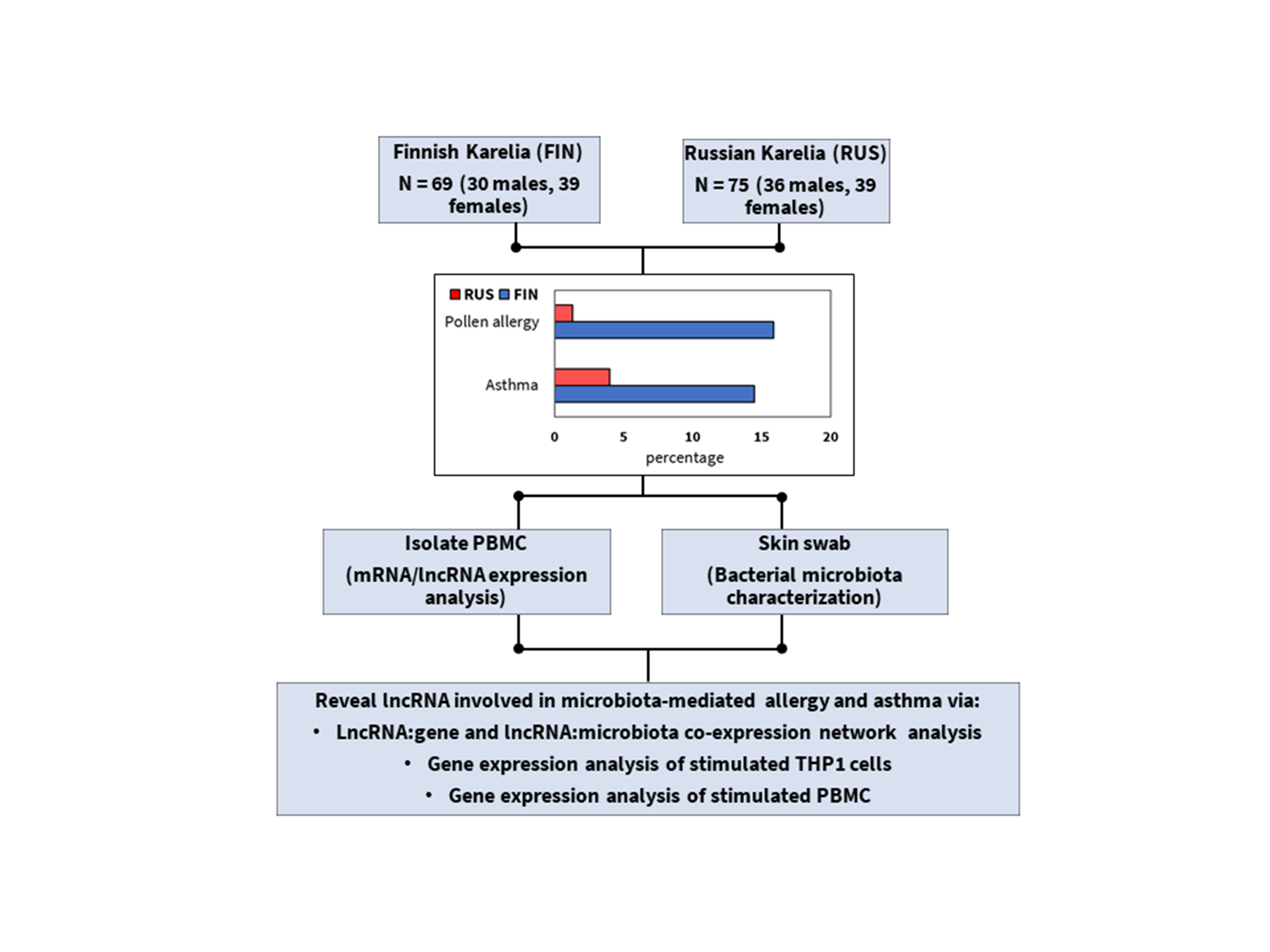

Supplement: Supplementary Figure S1 — Study design. Skin swab and whole blood samples derive from 69 Finnish and 75 Russian subjects, with marked differences in the prevalence of asthma and allergies. All 144 study subjects had paired skin microbiota data from skin swabs and transcriptomic data from peripheral blood mononuclear cells (PBMC). Correlation to protein-coding gene (mRNA) expression and microbiota abundance was used to predict long non-coding RNAs (lncRNA) involved in microbiota-immune interactions in asthma and allergy. Selected lncRNAs were validated with lipopolysaccharide and Betv1 allergen stimulations in a macrophage-like cell model (THP1) and donor-derived PBMC. [file Image_1.TIF]

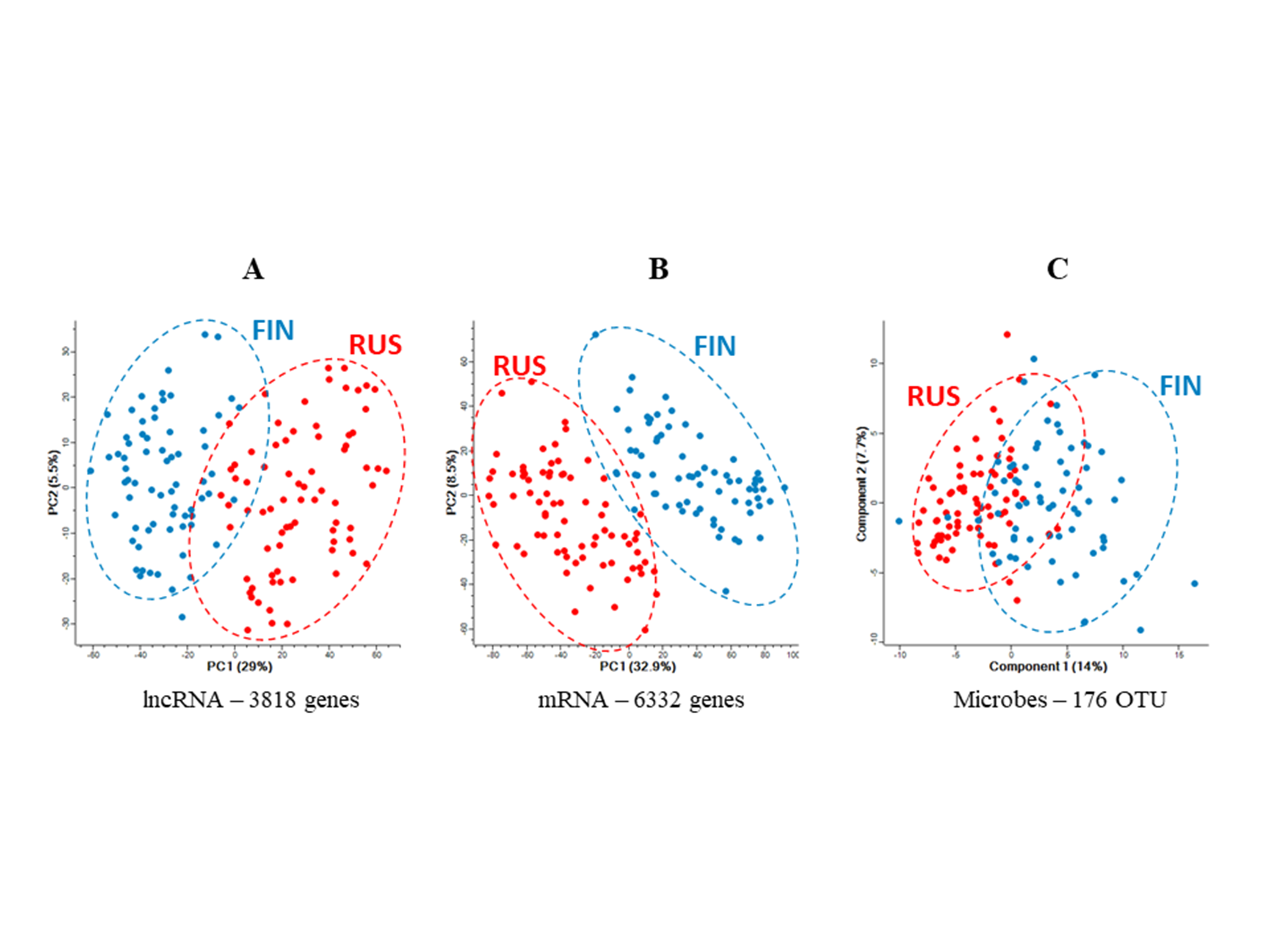

Supplement: Supplementary Figure S2 — Gene and microbiota-based distinction of Finnish and Russian samples. Principal component analysis of significantly different (adjusted p-value < 0.05) long non-coding RNAs [(A): lncRNA], protein-coding genes [(B): mRNA] and skin microbial taxa [(C): OTU]. All three data layers separate Finnish (FIN) from Russian (RUS) samples. In principal components 1 and 2, the most variation (41%) between the two populations was explained by differences in mRNA expression, followed by lncRNA expression (35%) and then microbiota abundance profiles (22%). [file Image_2.TIF]
